# The epidemiology of foot-and-mouth disease outbreaks and its history in Iraq

**DOI:** 10.14202/vetworld.2019.706-712

**Published:** 2019-05-25

**Authors:** Karima Akool Al-Salihi

**Affiliations:** Department of Internal and Preventive Medicine, College of Veterinary Medicine, Al-Muthanna University, Al-Muthanna, Iraq

**Keywords:** Baghdad, Bashder checkpoint, foot-and-mouth disease, Iraq, Nineveh governorate

## Abstract

**Background and Aims:**

Foot-and-mouth disease (FMD) is reported in Iraq since 1937 and occurs as a devastating seasonal epidemic. This study intended to explore the epidemiology of FMD in Iraq during 2011-2016, through assessment of outbreak reports among cow, buffalo, and small ruminants (sheep and goat) in 15 Iraqi governorates except for Kurdistan region.

**Materials and Methods:**

The reported data regarding FMD cases were collected from veterinary hospitals in 15 Iraqi governorates and were analyzed.

**Results:**

The results revealed annual FMD outbreaks in cow, buffalo, and small ruminants in Iraqi governorates with variability in the numbers of the infected and dead animals. The total number of infected animals increased in 2016 compared to 2015 due to the illegal importation of FMD-infected cows at the end of 2015. The prevalence rates of FMD were 68.7%, 46.6%, and 30.3% in cattle, buffalo, and small ruminants, respectively, in 2016, while this was 18.4%, 19.9%, and 17.3%, respectively, in 2015.

**Conclusion:**

This study approved the reemergence and endemic nature of FMD in Iraqi livestock. Prompt procedures and a new future strategy need to be implemented to control the increasing incidences of FMD in Iraq.

## Introduction

Foot-and-mouth disease (FMD) is an infectious disease that affects up to 70 species of cloven-hoofed mammals including cattle, sheep, goats, and pigs [1,2]. The causative agent of FMD virus (FMDV) belongs to the genus *Aphthovirus* and family *Picornaviridae*. FMDV is a small, non-enveloped, positive-sense, single-stranded RNA virus, with genome of approximately 8500 bases surrounded by four structural proteins which form an icosahedral capsid. There are seven immunologically distinct serotypes of FMDV (O, A, C, Asia 1, and Southern African Territories 1, 2, and 3), with a broad spectrum of antigenically and epidemiologically distinct subtypes within each serotype. The great diversity is considered a consequence of the high mutation rate, quasi-species dynamics, and recombination [3-6]. Many immunologically related subtypes between FMDV serotypes such as A5, A4, A24, C1, and O1 have been reported, totaling more than 60 type-subtype known combinations [7]. A genetic variation can occur via mutations or homologous recombination between two different strains of FMDV, which leads to generating new variants of FMDV. These new variants have significant implication for the selection of FMDV vaccine strains [8-10]. FMDV is highly contagious in nature and spreads rapidly through susceptible animals. The sporadic attack of disease into FMD-free regions is difficult and costly to control. FMD is most transboundary severe disease characterized by short incubation periods compared to any other infectious diseases. The disease causes fever and blister-like lesions or vesicles occurring on the oral cavity, nostrils, muzzle, coronary bands, teats, udder, and interdigital spaces. The results of these lesions are excessive salivation and lameness. In sheep and goats, the signs are usually less visible or mild and lead to difficulty in diagnosing the disease [11-13]. The morbidity rate approaches 100% in a susceptible animal. However, the disease is seldom fatal, except in young animals [14,15]. However, the disease causes huge economic damages due to loss of milk production in the 2 months after the beginning of the clinical signs [16].

FMD is endemic in Iraq and causes tremendous economic losses to owner’s livestock every year [2,9,12,17,18]. A review of literature revealed scarce publications regarding the epidemiology of FMD in Iraq. Moreover, there are appropriate plans for providing vaccine to the farmers, but there is limitation to implement it on the ground. Furthermore, the open retail is overwhelmed with the uncontrolled vaccine of undefined efficacy and free movement of the animal between different governorates in Iraq.

Therefore, this study intends to describe the status of FMD in Iraq, through assessment of FMD outbreak reports among cow, buffalo, and small ruminants in different Iraqi governorates during the last 6 years (2011-2016), apart from briefly reviewing the history of FMD outbreaks in Iraq.

## Materials and Methods

### Ethical approval

The study was approved by Animal and Research Ethical Committee, College of Veterinary Medicine, Al-Muthanna University, Iraq.

### Approach to the literature review

A literature search was done reviewing published journals, articles, Iraqi reports, and review articles. The search used the following method:

Online search including Iraqi Academic Scientific journals, PubMed, Google Scholar, Google Web, and Research Gate was undertaken for papers using medical subject heading terms Foot and mouth disease and Iraq as a significant entry and epidemiology as a subheading. The total search gave ten titles; however, particular categories of titles were excluded such as letters and editorials. The results of the search were restricted to Iraq, with articles in English or with English abstract. From the total search, 71 titles were retracted, and only 35 articles were kept for further analysis.

### Data collection and analysis

The reported data regarding FMD cases were collected from 15 veterinary hospitals of Iraqi governorate. Data were entered into Microsoft Excel. Categorical variables were described in percentages and 95% confidence intervals.

## Results

### History of FMD outbreaks in Iraq

The review of the literature resulted in only 35 publications consisting of 6 and 29 for international and Iraqi national publications, respectively. Although FMD has been reported since the late 19^th^ and the early 20^th^ centuries, the first notifiable case in Iraq was published in 1937 at the Sulimanyha/Bashder checkpoint Iraqi border in 202 cattle herd, where 11 of these cattle revealed progressive clinical signs of the disease. The first reported FMD outbreak in cattle and buffalo was observed in 1938, in Basra, Missan, and Diala, which are located on the Iraqi-Iranian borders. The residing wild boars in the border swampland in Basra and Missan played a dynamic role in the spread of the virus and transmission of the disease from Iran to Iraq [18]. These outbreaks occurred mainly in cattle and buffalo with total infected animals of 1109 (300 buffalo and 809 cattle), 324 (256 buffalo and 68 cattle), and 135 in Diala, Missan, and Basra, respectively. Meanwhile, caustic soda between 4/1000 and 8/1000 concentrations was used to disinfect the hooves of the infected animals and contaminated objects in those areas to enclose the outbreak. The disease was also reported in 1945, as outbreak between buffalo, cattle, sheep, and goat, with about 20,000 infected animals and 113 dead animals. Meanwhile, about 53,094 animals were vaccinated during this time. Then, in 1957, FMD occurred in buffalo, cattle, sheep, and goats as large outbreaks comprising most of the Iraqi governorates, with 801,135 infected animals. Then, caustic soda was used to decrease the symptoms and reduce economic losses. Later on, FMD continued to affect livestock in Iraq as an outbreak. The disease continued to be recorded in the 1960s, 1970s, 1980s, and 1990s. The 1998/1999 epidemic is considered the most severe FMD outbreak that occurred during the economic sanction on Iraq. It led to a huge loss in cattle and small ruminants in 13 Iraqi governorates. The total number of infected animals was 50,678 and 982,309 for cattle and small ruminants, respectively. Moreover, the mortality rate was 7.5% (3,832) and 4.9% (48,089) for cattle and small ruminants, respectively [19]. According to El Idrissi [20], the 1998 FMD outbreaks affected about 25% of the ruminant population (approximately three million ruminants with massive losses in newly born animals). The subserotype O1 Middle East was the primary causative agent. The predicted number of dead animals was 500,000 due to the poor veterinary service during the period of economic sanction and lack of proper preparations accompanied by free animal movement. All these reasons were attributed to the deterioration of the condition and led to the difficulties in supplying vaccine for the farmers and raising the mortality rate.

Consequently, after the Sanctions Committee authorized deliveries of FMD vaccines to Iraq, the vaccines were provided, and a bulk vaccination platform was introduced to enclose the disease. The effect of 1998/1999 FMD outbreak continued until 2000-2001. After that, no record of notifiable FMD infection was reported due to the implementation of vaccination campaigns after 1999, wherein more than 27 million small and large ruminants were vaccinated. In 2003, the third Gulf War started, which led to the interruption in most public and governmental services including the damage of veterinary service centers in the majority of Iraqi governorates. Moreover, the open Iraqi borders with neighboring countries, a free smuggler of livestock, heavy entry of uncertified animal products, sectarian violence, and lack of veterinary quarantine all contributed to the reemerging of FMD in 2004. A total of 967 cattle and buffalo and 2315 small ruminants were infected. In 2006, a new FMDV serotype A lineage was transmitted from neighboring Iraqi countries due to uncontrolled livestock and animal product movement, leading to episodes of infection in Iraq. The number of infected animals increased in 2007 to reach 12,957 in goats and sheep and 2828 in cattle in all governorates. A substantial decline in the number of infected animals was reported in 2008. In early 2009, 148 outbreaks reported to OIE from different provinces with infection of 1612 sheep and goats, 779 cattle, and 19 buffaloes. There were 19 deaths. The devastating 2009 FMD continued to infect more than 14,036 and 14,198 affected cases in sheep and goat and cattle and buffalo, respectively, in all Iraqi governorates. FMD continued as an epidemic in 2010 due to the delayed processing in releasing of vaccine from the veterinary service accompanied by the absence of management and coordination.

### Serotypes of FMDV reported in Iraq during decays of outbreaks

In Iraq, serotype A was reported officially for the 1^st^ time in 1952 according to FAO/FMD reference laboratory (IAH, Pirbright Laboratory). Later on, different serotypes were determined such as Asia 1 in 1975, 1983, and 1984; O1 in 1985 and 1993; O Manisa in 1998 and 1999; and A IRN 96 in 2000 and 2002 [12]. The World Reference Laboratory for FMD reported that a new subtype of FMDV serotype A was detected in Iran in 2005. This serotype was responsible for substantial disease outbreaks in the Middle East that distributed rapidly throughout Iran and Saudi Arabia in 2006, Turkey in 2005, and Jordan in 2007. This serotype designated as A/IRN/2005 [14] was also reported in Iraq from different samples collected from FMD outbreaks in various Iraqi regions during 2009, 2010, 2011, 2012, and 2013, when these samples were sent to the FAO/FMD reference laboratory (IAH, Pirbright Laboratory) for genotyping and phylogeny reconstruction. Different FMDV serotypes were determined, namely FMDV-A ASIA Iran05 BAR-08, FMDV-O ME-SA PanAsia2 ANT-10, FMDV-A ASIA Iran05 AFG-07, FMDV-A Asia 1 Sindh-08, and FMDV-A Asia1 Iran05SIS-10 (Table-1) (Donald King/Head of WRLFMD, The Pirbright Institute, UK, 2016) [21].

**Table-1:** FMDV serotypes reported in Iraq since 1957.

Serotype	Years of record
FMDV-O	1957, 1959, 1961, 1964, 1966, 1969, 1970, 1973, 1977, 1985, 1994, 1999, 2001, 2010
FMDV-A	1952, 1955, 1963, 1964, 1970, 1978, 1985, 2000, 2002, 2009, 2010, 2012, 2013
FMDV-Asia1	1975, 1985, 2011, 2012
FMDV-SAT1	1962
FMDV-A ASIA Iran05^BAR-08^	2009
FMDV-A ASIA Iran05^AFG-07^	2010
FMDV-O ME-SA PanAsia2^ANT-10^	2011
FMDV-A Asia 1 Sindh-08, FMDV-A Asia1 Iran05^SIS-10^	2013

Source: (FAO World Reference/Laboratory for Foot and Mouth Disease) http://www.wrlfmd.org/fmd_genotyping/me/irq.htm. ([FAO World Reference/Laboratory for Foot and Mouth Disease], n.d.), FMDV=Foot-and-mouth disease virus

### Epidemiology of FMD in Iraq between 2011 and 2016

The disease started to reemerge in 2011, where the whole infected animals were 6757, 5216, and 13,305 of cattle, buffalo, and small ruminants, respectively. While, the number of dead animals was 147, 39, and 1293 of cattle, buffalo, and small ruminants, respectively. Meanwhile, it was predicted that the number of infected and dead animals did not reflect the actual situation due to the lack of epidemiological investigation, reporting, and veterinary facilities. During 2011, the veterinary service started vaccination campaign and vaccinated around 1,935,510 and 7,062,003 of cattle and buffalo and small ruminants, respectively.

In 2012, FMD outbreaks reemerged in 15 Iraqi governorates but with a decline in the number of infected animals. There were 2336, 125, and 1523 infected cattle, buffalo, and small ruminants, respectively, while the number of dead animals was 73, 6, and 100 for cattle, buffalo, and small ruminants, respectively. Besides, during 2012, the veterinary service started vaccination campaign and vaccinated more than 1,798,074 and 7,105,941 cattle and buffalo and small ruminants, respectively.

In 2013, 2014, and 2015, an obvious decline in the number of FMD-infected animals was observed. The number of infected cattle was 467, 25, and 68; buffalo 609, 15, and 763; and small ruminant 675, 89, and 110 for 2013, 2014, and 2015, respectively. In addition, the number of vaccinated animals was 1,842,385; 1,736,437; and 1,078,231 for cattle and buffalo and 7,105,941; 4,455,739; and 2,196,460 for small ruminants, for the years 2013, 2014, and 2015, respectively.

**Figure-1:**
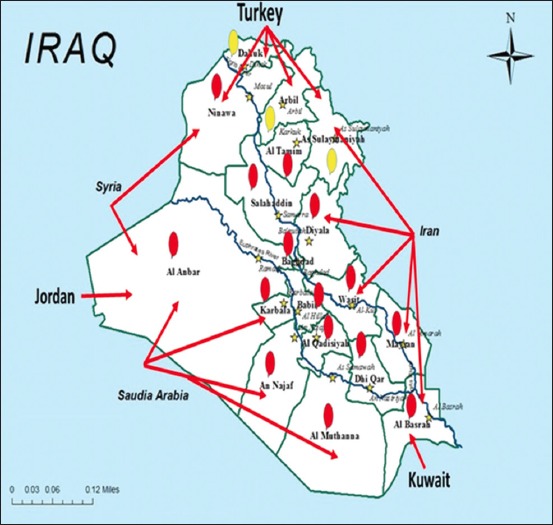
Map of Iraq (Source: Iraq map template from en.wikipedia.org) showing the foot-and-mouth disease (FMD) outbreaks in Iraq in 2016. Yellow stars=Provincial capital; Red balloon=FMD infected foci in different governorates, Yellow balloon=Excluded governorate, red arrow=The expected source of the movement of FMD virus infection from the neighboring countries.

**Figure-2:**
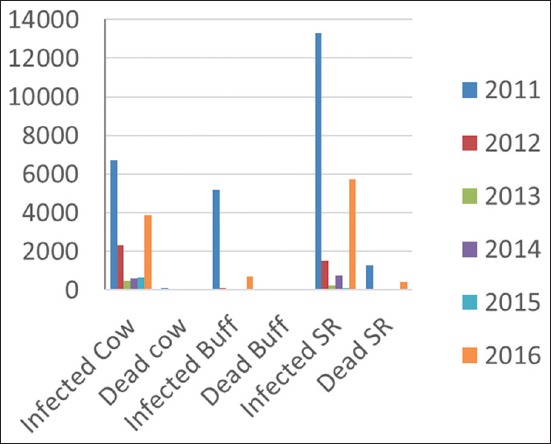
The total number of foot-and-mouth disease infected and dead animals during 2011, 2012, 2013, 2014, 2015, and 2016. (SR=Small ruminant).

**Figure-3:**
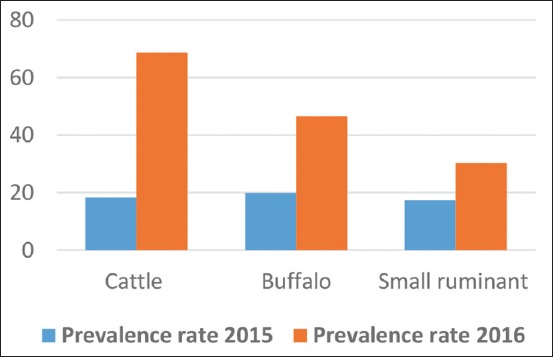
The prevalence rate of foot-and-mouth disease-infected animals in 2015 and 2016.

**Figure-4:**
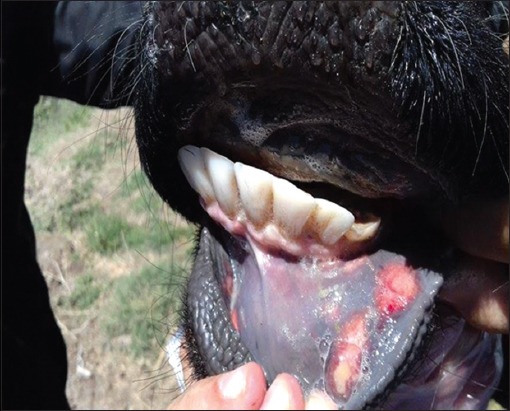
Typical foot-and-mouth disease oral lesions (ulceration after rupture of blisters) during 2016 outbreak.

In 2016, a noticeable increase was seen in the number of FMD-infected animals in 15 Iraqi governorates (Baghdad, Karbala, Najaf, Dewania, Al-Muthanna, Thiqar, Missan, Basra, Ninawa, Kirkuk, Salah-Eldin, Al-Nabar, Diala, Wassit, and Babil) (Figure-1). The number of recorded cases was 3891, 782, and 5743 in cattle, buffalo, and small ruminants, respectively. While the number of dead cows, buffalo, and sheep and goat was 44, 0, and 457, respectively (Figure-2). According to veterinary service, the prevalence rate increased in 2016 in comparison to 2015 in cattle, buffalo, and small ruminants (Table-2 and Figure-3). The infected animals showed the classic symptoms and lesions of FMD. The suspected animals revealed salivation, blisters, and ulcers on the gums and lips as well as on the feet (Figure-4). The veterinary service started the vaccination campaign, and the number of vaccinated animals was 1,541,375 cattle and buffalo and 4,833,721, respectively (Table-3). Although Iraqi veterinary market was flooded with an unauthorized vaccine of suspicious efficacy due to open trade, recently, the veterinary service has used the imported vaccine from FEDERAL CENTER FOR ANIMAL HEALTH (FGBI “ARRIAH” [Yur′ evets, Vladimir, Russia]). This vaccine is called “ARRIAH”-VAC and composed of O/Tur/5//2009, A/Tur/06-20, and Asia 1/Pak/08 Sindh-8 types.

**Table-2:** The prevalence rate of foot-and-mouth disease in animals in 2015 and 2016.

Animal	Year 2015 (%)	Year 2016 (%)
Cattle	18.4	68.7
Buffalo	19.9	46.6
Small ruminant	17.3	30.3

**Table-3:** The total number of vaccinated animals in Iraqi veterinary service campaign during 2011-2016.

Year	Number of vaccinated cattle and buffalo	Number of vaccinated sheep and goat
2011	October-November 1,935,510 (68.2%)	November-December 7,062,003 (76.78%)
2012	April-June 1,798,074 (63.36%)	May-June 7,003,891 (76.87%)
2013	January 1,842,385 (64.9%)	January 7,105,941 (77.2%)
	May-July 1,710,533 (60.27%)	May-July 6,962,684 (75.7%)
		September-November 6, 962,684 (94.27%)
2014	April-June 1,736,437 (89.25%)	September-December 4,455,739 (102.69%)
	September-December 1,307,954 (83.11%)	
2015	January-March 1,078,231 (73.77%)	January-March 2,196,460 (53.03%)
2016	April 1,705,033 (61.27%)	May 2,096,460 (50.03%)

## Discussion

FMD is a highly contagious viral disease. Furthermore, it is a severe transboundary animal infection, with rapid and predicted national and international spread. FMD can cause disabling socioeconomic consequences, due to its high impact on production and trade. Ruminants have an ability to harbor the FMDV in their pharyngeal tissues for an extended period that occurs when recovered or vaccinated cattle expose to diseased animal. Moreover, these animals can become healthy carriers for 3-5 years. Moreover, sheep can be carriers of the virus for 4-6 months, whereas pigs are not a carrier [22,23].

Iraq is the land of Mesopotamia that has domesticated small and large ruminants for more than 6000 years. Iraq is rich in livestock and is considered the nucleus of all species of farm animals. The Iraqi Ministry of Agriculture conducted the census of animals in 2008, and the estimated population of cattle, buffalo, sheep, goat, and camel was 2,552,113; 285,537; 7,722,375; 1,474,846; and 58,293, respectively [24]. However, the comparison of these numbers with 1978 animal’s census revealed a drop in animal’s population, where the estimated numbers were 9.7, 2.1, 1.7 million and 70,000 for sheep, goat, cattle, and camel, respectively, except for buffalo [25]. After 2003, the border control between Iraq and neighboring countries was corrupted down and assisted in the smuggling of different types of animals from Iraq to the outside and *vice versa* that led to various serious transboundary diseases, such as FMD and lumpy skin disease [25,26]. All countries of the Middle East are continuously suffering from the devastating epidemics of FMD that distributes rapidly across the country’s borders. Geographically, Iraq is situated in the heart of the Middle East, and this location is indeed affected by the FMD epidemiological situation of the neighboring countries. It is important to study and identify the reemergence of FMD in Iraq because it is a contiguous disease. The sectarian violence and aggressive rules in Iraq and Syria in 2014 and the consequences led to the breakdown of all government services and activities, particularly in veterinary services in the parts of Iraq that came out of Iraqi government control (Nineveh, Salah Al-Din, and Anbar), leading to wide ranging interruption of livestock vaccination programs. Moreover, the uneventful situations resulted in open borders and massive smuggler processes of the livestock with neighboring countries accompanied by absence and/or loss of veterinary quarantine procedures. In addition, this situation made Iraq as an open market place for animal products without authorization or licensed veterinary certificate.

According to the FAO-UN monthly report on FMD situation in 2016, Iraq was included in the West Eurasia and the Middle East countries according to its geographical location that has similarities with the ecosystem of virus pool 3 and dominated with FMDV serotypes O, A, and Asia 1 [21].

The current study revealed the recurrent appearance of FMD outbreaks in Iraq during 2011 consequence to the severe outbreaks that occurred during 2009-2010 and led to the devastating economic loss in the livestock in Iraq. In 2010, more than 130 outbreaks were recorded in all Iraqi governorate constituent; 14.036 and 14,198 infected cases in sheep and goat and cattle and buffalo, respectively. There are several reasons for the reemergence cases which led to continuity of FMD as an epidemic in 2010; first of all, the delayed processing in releasing vaccine with the absence of management and coordination. At the end of 2010, 2-time yearly vaccination campaign was started; accordingly, the total number of the new FMD cases drastically decreased to less than half in 2011. Additionally, there was an apparent drop in the total number of newly infected cases in 2012, 2013, 2014, and 2015 due to the continuous vaccination program.

Due to the illegal importation of FMD-infected cows from one of the neighboring countries via Basra/Shat Al-Arab seaport at the end of 2015, livestock in 15 Iraqi governorates suffered from FMD infection in early 2016. The disease occurred as outbreaks and revealed a high prevalence rate in cattle, buffalo, and small ruminants. All animals showed the typical symptoms and lesions of FMD. There are several predisposing risk elements for the annual reemergence of FMD in Iraq, and the principal compelling risk reason is free trade of live animals much higher than meat because traditionally, the Iraqi people prefer consumption of meat from the slaughter of live animals despite the absence of proper disposal of carcasses. The continuous introduction of small ruminants in the Kingdom of Saudi Arabia, especially during Muslim festivals in Hajj (Eid-Adha), led to the rapid distribution of a new serotype that emerged in other regions.

In this decade (the 2010 decade), new serotypes were reported in Iraq such as FMDV-A ASIA Iran05 BAR-08, FMDV-A ASIA Iran05 AFG-07, FMDV-O ME-SA PanAsia2 ANT-10, and FMDV-A Asia 1 Sindh-08 and FMDV-A Asia1 Iran05SIS-10 in 2009, 2010, 2011, and 2013, respectively. There is the probability that a new sublineage of virus led to 2016 FMD outbreak in Iraq with increased number of infected animals. Moreover, the reason was probably due to use of the vaccine strain that did not closely match the isolated serotypes from the field. This interpretation is in agreement with Elnekave *et al*. [27] and Kitching *et al*. [28] who mentioned that the vaccine would influence the value of FMD control by vaccination strains available and at this time, closely matched serotypes would be required immediately to formulate a vaccine. During 2009, the veterinary service in Iraq implemented the vaccination strategies to control FMD. Two types of vaccines, namely trivalent (O, A22, and Asia 1) and a monovalent vaccine (O Manisa) were used for buffalo and cattle and sheep and goats, respectively [29-31]. The 2009 devastating FMD outbreaks continued in 2010-2011, and the number of infected animals declined in the subsequent years 2012-2015 after the vaccination campaign. However, this vaccine became non-protective at the end of 2015 and led to reemergence of FMD in early 2016. With these events, the Iraqi veterinary service started to use the “ARRIAH”-VAC formulated from O/Tur/5//2009, A/Tur/06-20, and Asia 1/Pak/08 Sindh-8 types that match the isolated serotypes from the Iraqi field. During any disease epidemics, the early detection of the disease plays a fundamental role in the success of preventing and eradicating the epidemic before causing extensive damage. Furthermore, the quick initial reaction will help in reducing the influence of FMD on animal health and economy. The distribution of new FMD outbreak routinely occurs when there is failure in the surveillance and disease-reporting plan in the country.

Moreover, the shortage in the vaccine provision and absence of information exchange and active communication with neighboring countries will worsen the situation. Iraq has implemented the FMD control program since the 1970s, and Al Dora FMD laboratories were established in the early 1970s. These laboratories were used for the identification of various strains of the virus that cause the disease. Moreover, it was a high producer of triple vaccine (Serotype A, O, and Asia 1) that met all Iraq’s needs and surrounding areas and helped in the success of the FMD control plan in Iraq and the Middle East. However, the second Gulf War (1991) and the economic sanction on Iraq led to suspension of work, disabling the FMD vaccine production that led to weakening of the FMD control program in Iraq as well as in the Middle East. These events contribute to the continuity of the threat of FMD on the livestock and the national economy. Therefore, reassessment of FMD control program is necessary at this stage.

Moreover, the success of the FMD control program requires adequate knowledge about FMDV and disease epidemiology. Sufficient veterinary infrastructure and resources including veterinary field staff and adequate diagnostic and research facilities are essential to achieve the goal. Furthermore, appropriate legislation is required to control the animal movement and compulsory vaccination, apart from sufficient budget, to implement an effective control program.

## Conclusion

FMD is an endemic disease in Iraq and periodically occurs as a devastating epidemic since 1937. Due to continuous war and political and sectarian conflicts in Iraq, the veterinary service and infrastructure severely weakened the disease control. The open Iraqi borders and uncontrolled animal movements led to introduction of exotic FMDV strains. Besides, the poor FMD observation and emergency awareness play the fundamental components in fighting the endemic disease. The restricted diagnostic requirements and the obstacles in hampering animal movement and absence/or unbalanced source of suitable vaccines are considered the primary tasks challenging the employment of a successful and efficient FMD control system in Iraq. Iraq also should take the awareness about the new serotype A lineage (named A/ASIA/G-VII [G-18]) that has currently emerged from the Indian subcontinent and is now rapidly spreading in the Middle East and presently detected in Saudi Arabia, Iran, Armenia, and Turkey. The FMD condition in Iraq creates a threat to the neighboring countries and worldwide, and *vice versa*. Strengthened effort and management from Iraqi veterinary service is essential to restrict the disease and its many significant consequences.

## Authors’ Contributions

The author collected and analysed the data, drafted and revised the article.
